# Enhancing the prediction of acute kidney injury risk after percutaneous coronary intervention using machine learning techniques: A retrospective cohort study

**DOI:** 10.1371/journal.pmed.1002703

**Published:** 2018-11-27

**Authors:** Chenxi Huang, Karthik Murugiah, Shiwani Mahajan, Shu-Xia Li, Sanket S. Dhruva, Julian S. Haimovich, Yongfei Wang, Wade L. Schulz, Jeffrey M. Testani, Francis P. Wilson, Carlos I. Mena, Frederick A. Masoudi, John S. Rumsfeld, John A. Spertus, Bobak J. Mortazavi, Harlan M. Krumholz

**Affiliations:** 1 Center for Outcomes Research and Evaluation, Yale-New Haven Hospital, New Haven, Connecticut, United States of America; 2 Section of Cardiovascular Medicine, Department of Internal Medicine, Yale School of Medicine, New Haven, Connecticut, United States of America; 3 Robert Wood Johnson Foundation Clinical Scholars Program, Department of Internal Medicine, Yale School of Medicine, New Haven, Connecticut, United States of America; 4 Veterans Affairs Connecticut Healthcare System, West Haven, Connecticut, United States of America; 5 Albert Einstein College of Medicine, Bronx, New York, United States of America; 6 Department of Laboratory Medicine, Yale School of Medicine, New Haven, Connecticut, United States of America; 7 Section of Nephrology, Department of Internal Medicine, Yale School of Medicine, New Haven, Connecticut, United States of America; 8 Division of Cardiology, School of Medicine, University of Colorado, Aurora, Colorado, United States of America; 9 Department of Cardiology, Saint Luke’s Mid America Heart Institute, Kansas City, Missouri, United States of America; 10 Department of Computer Science & Engineering, Texas A&M University, College Station, Texas, United States of America; 11 Department of Health Policy and Management, Yale School of Public Health, New Haven, Connecticut, United States of America; University of Oxford, UNITED KINGDOM

## Abstract

**Background:**

The current acute kidney injury (AKI) risk prediction model for patients undergoing percutaneous coronary intervention (PCI) from the American College of Cardiology (ACC) National Cardiovascular Data Registry (NCDR) employed regression techniques. This study aimed to evaluate whether models using machine learning techniques could significantly improve AKI risk prediction after PCI.

**Methods and findings:**

We used the same cohort and candidate variables used to develop the current NCDR CathPCI Registry AKI model, including 947,091 patients who underwent PCI procedures between June 1, 2009, and June 30, 2011. The mean age of these patients was 64.8 years, and 32.8% were women, with a total of 69,826 (7.4%) AKI events. We replicated the current AKI model as the baseline model and compared it with a series of new models. Temporal validation was performed using data from 970,869 patients undergoing PCIs between July 1, 2016, and March 31, 2017, with a mean age of 65.7 years; 31.9% were women, and 72,954 (7.5%) had AKI events. Each model was derived by implementing one of two strategies for preprocessing candidate variables (preselecting and transforming candidate variables or using all candidate variables in their original forms), one of three variable-selection methods (stepwise backward selection, lasso regularization, or permutation-based selection), and one of two methods to model the relationship between variables and outcome (logistic regression or gradient descent boosting). The cohort was divided into different training (70%) and test (30%) sets using 100 different random splits, and the performance of the models was evaluated internally in the test sets. The best model, according to the internal evaluation, was derived by using all available candidate variables in their original form, permutation-based variable selection, and gradient descent boosting. Compared with the baseline model that uses 11 variables, the best model used 13 variables and achieved a significantly better area under the receiver operating characteristic curve (AUC) of 0.752 (95% confidence interval [CI] 0.749–0.754) versus 0.711 (95% CI 0.708–0.714), a significantly better Brier score of 0.0617 (95% CI 0.0615–0.0618) versus 0.0636 (95% CI 0.0634–0.0638), and a better calibration slope of observed versus predicted rate of 1.008 (95% CI 0.988–1.028) versus 1.036 (95% CI 1.015–1.056). The best model also had a significantly wider predictive range (25.3% versus 21.6%, *p* < 0.001) and was more accurate in stratifying AKI risk for patients. Evaluated on a more contemporary CathPCI cohort (July 1, 2015–March 31, 2017), the best model consistently achieved significantly better performance than the baseline model in AUC (0.785 versus 0.753), Brier score (0.0610 versus 0.0627), calibration slope (1.003 versus 1.062), and predictive range (29.4% versus 26.2%). The current study does not address implementation for risk calculation at the point of care, and potential challenges include the availability and accessibility of the predictors.

**Conclusions:**

Machine learning techniques and data-driven approaches resulted in improved prediction of AKI risk after PCI. The results support the potential of these techniques for improving risk prediction models and identification of patients who may benefit from risk-mitigation strategies.

## Abstract 2

In a retrospective cohort study, Harlan M. Krumholz and colleagues use machine learning techniques in an effort to improve identification of PCI patients who may benefit from AKI risk-mitigation strategies.

## Author summary

**Why was this study done?:**

- Accurately estimating the risk of developing acute kidney injury (AKI) is important to determine revascularization strategies and inform peri- and postprocedural care.
- The current AKI risk prediction model was developed by employing prior clinical knowledge to choose and transform candidate variables and applying regression techniques.
- It is unknown whether and how machine learning techniques could improve predictive performance from the current AKI risk model.

**What did the researchers do and find?:**

- We used data from 947,091 percutaneous coronary intervention (PCI) procedures from the National Cardiovascular Data Registry (NCDR) CathPCI registry to develop AKI prediction models, the same data from which the current AKI risk model was derived. Temporal validation was performed on a more contemporary cohort of 970,869 PCIs from the NCDR CathPCI registry.
- We compared the performance of the current AKI risk model with a series of new AKI risk models, which were derived using different regression and machine learning techniques.
- An AKI risk model derived from machine learning techniques had significantly better discrimination, calibration, and risk stratification than the current AKI risk model in both the internal test set and temporal validation set.

**What do these findings mean?:**

- The demonstrated improvement in predictive power supports the potential of machine learning techniques for improving risk prediction modeling.
- The improved prediction for AKI suggests great potential of the model to improve targeting of risk-mitigation treatment and quality assessment of cardiovascular care.
- Future studies need to assess the feasibility of integrating the machine learning model into clinical care and evaluate the benefit of the model for patients at the point of care.

## Introduction

Acute kidney Injury (AKI) is an important complication of percutaneous coronary intervention (PCI) and is associated with increased in-hospital and long-term morbidity and mortality [1,2]. Accurately estimating patients’ risk of developing AKI is important for physicians in order to determine revascularization strategies, as well as to inform peri- and postprocedural care [3–5]. Specifically, being able to identify patient risks can inform personalized strategies to minimize contrast-induced nephropathy [6]. Moreover, identifying an individual patient’s risk can support shared decision-making regarding clinical strategies that may involve PCI.

An AKI risk prediction model was recently developed for patients undergoing PCI, using data from the American College of Cardiology (ACC) National Cardiovascular Data Registry (NCDR) CathPCI registry [7]. This widely used model was developed following traditional strategies for building prediction models in medicine, including preselecting and transforming candidate variables based on prior knowledge, applying hierarchical logistic regression to model the relationship between variables and outcome, and, finally, reducing the number of variables via stepwise selection for a more parsimonious model to support prospective application. Some potential nonlinearity and interactions were tested, but none were significant [7]. These traditional approaches to risk stratification depend on subjective assumptions to choose candidate variables and often transform them into categorical variables for convenience when calculating risk scores, which may reduce the available information and miss unexpected relationships that could be leveraged to aid prediction. Further, there is potential for model performance to benefit from other variable-selection methods than the most frequently implemented stepwise selection. The stepwise selection is known to have the drawback of being unstable, as different variables may be selected with small changes in the data [8–10]. Finally, even though nonlinear relationships and interactions can be modeled to a degree through logistic regression, they must be defined explicitly prior to building the model, and it may be infeasible to exhaustively test all possible nonlinearity relationships and interactions when the number of candidate variables is large. As a result, higher-order interactions are mostly ignored, even though consideration of these interactions may improve model performance.

An alternative strategy for developing risk estimation models is to use machine learning–based methods such as tree-based models, which do not require assumptions regarding the variables and their relationships with the outcome. Therefore, these techniques can incorporate complex relationships in a completely data-driven manner, including nonlinearity or interactions that may be hard to detect by regression-based models [11–15]. There is thus the potential to improve AKI risk prediction by including machine learning methods as part of the model development strategy.

To explore the machine learning techniques in risk prediction, we sought to determine whether a model for predicting AKI risk derived from these techniques would yield higher predictive accuracy than the current AKI model. Not only did we seek to build and compare machine learning models with regression techniques, but we also sought to provide insight into how the improvement was achieved. To do so, we used the same cohort and candidate variables from the NCDR CathPCI registry for developing the current AKI model with the use of 11 preprocedural variables, and, with this cohort, we developed and validated a series of new models, derived using different methods. By comparing the performance of these new models with the current AKI model, we attempted to answer 3 questions: First, using the same candidate variables, can other variable-selection techniques, including machine learning–based methods, produce better models than the stepwise selection? Second, using the same set of variables, could machine learning models improve performance over logistic regression through their capacity to capture complex relationships between these variables and outcome? Finally, could model performance be improved by availing more candidate variables in their original form to build the model, reducing the amount of subjective preselection or transformation of the variables? To gain further understanding of the benefits of the new best-performing model over the current AKI model for individual patients, we examined the range of predicted risks and the accuracy of stratifying risks of patients by the two models. We also validated our findings on the most contemporary data from the registry.

## Methods

We used data from the NCDR CathPCI registry to determine if the current AKI prediction model, which used 11 preprocedural variables for prospective risk estimation in routine clinical care, could be improved by using different approaches for preprocessing, variable selection, and relationship modeling, including the use of tree-based machine learning methods. The current AKI model was the baseline model to which the newly developed prediction model was compared. For all models, including the baseline model, we used the same dataset, candidate variables, and approach to handling missing data. This study is reported as per the transparent reporting of a multivariable prediction model for individual prognosis or diagnosis (TRIPOD) guidelines (S1 Checklist).

### Data source and study population

The NCDR CathPCI registry has been previously described [16,17]. Briefly, this registry includes data on patient characteristics, clinical presentation, treatments, and outcomes associated with PCI at more than 1,500 participating sites across the United States. Data are monitored through a comprehensive data quality program [18].

We applied the same inclusion and exclusion criteria to replicate the cohort used to develop the baseline model. Specifically, we included all PCI procedures between June 1, 2009, and June 30, 2011—the same period in which the baseline model was developed and tested (*n* = 1,254,089, S1 Fig). We excluded PCIs that were not the first during a single hospitalization (*n* = 32,999), procedures with same-day discharge (*n* = 41,570), missing serum creatinine before or after the procedure (*n* = 208,158), and procedures on patients already on dialysis at the time of their PCI (*n* = 24,271). The final analytic cohort included the remaining 947,091 PCI procedures. We restricted our analytic cohort to the same time period used for the baseline model development to rule out the potential impact of newly acquired data on model performance and changes in the importance of predictors when replicating the baseline model. To ensure generalizability of our results to contemporary practice, we also used a contemporary cohort that contained a total of 970,869 PCI procedures between July 1, 2015, and March 31, 2017, by implementing the same exclusion criteria as in the baseline model. The analysis was approved by the Institutional Review Board at Yale University.

### Study outcome

Following the same definition used in developing the baseline model, the outcome is post-PCI AKI, defined by the Acute Kidney Injury Network as the change of peak serum creatinine before and after the procedure [19]. A change in postprocedure creatinine larger than 0.3 mg/dL or a 1.5-fold increase from before the procedure is defined as AKI.

### Candidate variables

Following the same approach as the baseline model development, we considered potential predictor variables to be those available prior to the initiation of PCI. Accordingly, from Version 4.4 of the NCDR CathPCI Registry data collection form [20,21], we extracted variables from patient demographics, admission source, medical history and risk factors, clinical evaluation leading to the procedure (not including results of stress or imaging studies), mechanical ventricular support (including intra-aortic balloon pump) at the start of the procedure, PCI status and pre-PCI left ventricular ejection fraction (LVEF), creatinine, and hemoglobin. We extracted 50 variables that were strictly preprocedural and had a missing rate less than 3%, except for pre-PCI LVEF, which had a 29.4% missing rate (S1 Table). These variables were considered as the initial set of candidate variables for all models developed in this study, including the baseline model from which the authors further reduced the number of candidate variables, as described in the Model development techniques section. Following the same strategy used in the baseline model development, missing variables were imputed by the most common value for categorical variables and median for continuous variables. The full list of candidate variables with corresponding variable names and identifiers in the data collection form can be found in S1 Table.

### Model development techniques

**Fig 1:**
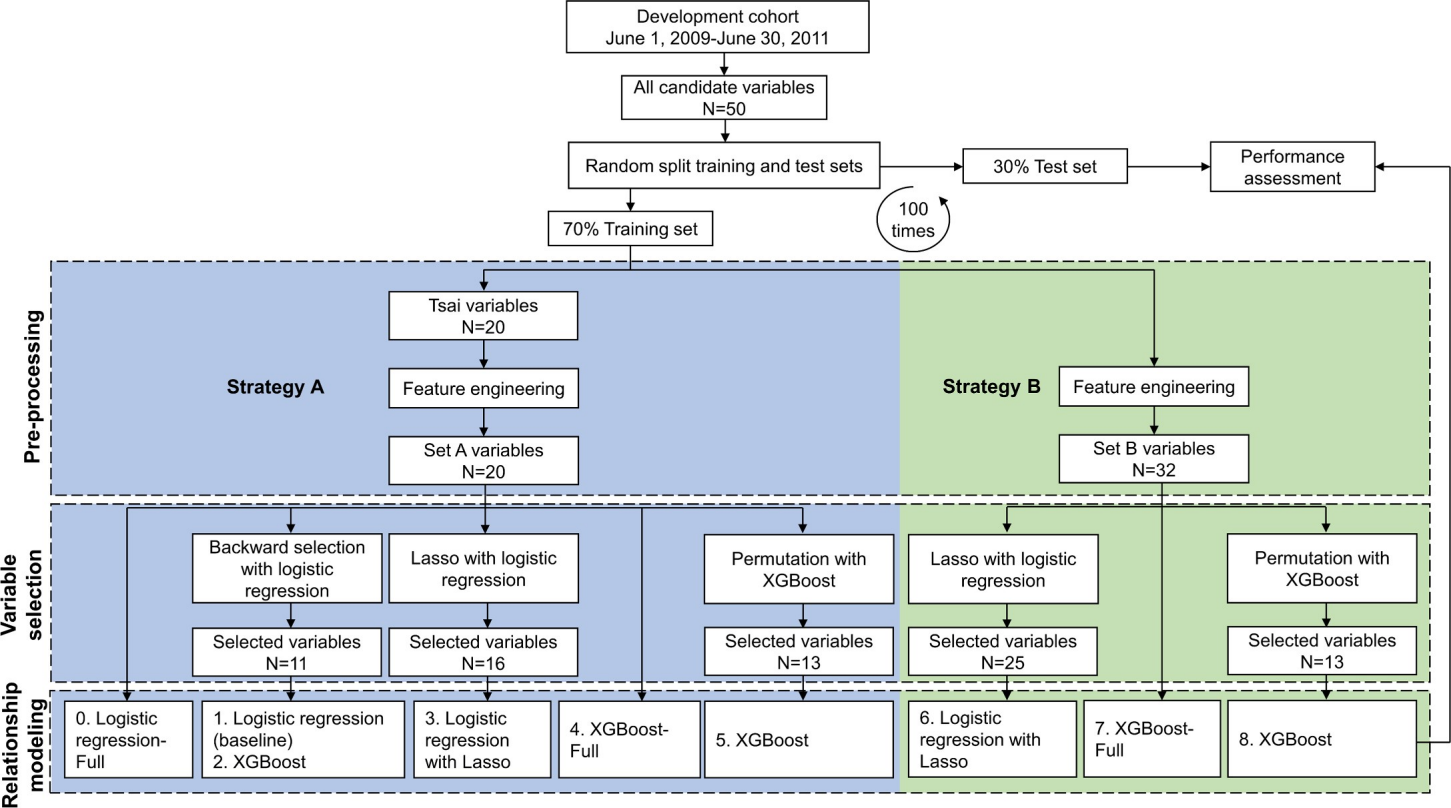
Analysis flow for developing and evaluating models.

The development of prediction models can be categorized into 3 major stages: preprocessing, variable selection, and relationship modeling (Fig 1, dashed boxes). Decisions on methods used in these stages can lead to prediction models with different performance. In the following, we describe the different methods we implemented, including both regression and machine learning techniques, in each of the 3 stages and the resulting prediction models we developed for performance comparison. These models were developed to test each of our 3 study objectives.

#### Preprocessing

The preprocessing stage includes the optional step of choosing candidate variables and the step of feature engineering. Traditional strategies of building prediction models often include a step to generate, from all candidate variables, a shorter list of variables that are deemed potentially predictive for the outcome, informed through statistical analysis as well as prior knowledge and clinical judgement. During the feature engineering step, the original variables may be transformed, which may include, but is not restricted to, converting continuous variables to categorical variables, normalizing variables to proper scales, and combining multiple variables into single variables for information integration.

We considered 2 strategies during preprocessing (Fig 1). Strategy A is the same as that implemented in the baseline model. When developing the baseline model, the authors chose 20 variables from all candidate preprocedural variables during preprocessing, based on clinical or statistical association with the outcome (Fig 1, Strategy A). Further feature engineering was applied to some of these variables to form the resulting Set A variables that were used for subsequent modeling (S2 Table). In contrast, Strategy B directly applied feature engineering to all candidate variables without imposing the discretionary decisions made in constructing the baseline model. Only information integration was performed in Strategy B. An example of information integration is combining the diabetes variable that takes values of “yes” or “no” and the diabetes therapy variable for those who responded “yes” to the diabetes variable. In this case, the 2 variables were combined to a composite variable that takes the following values: “Not diabetic,” “Diabetic and oral therapy,” “Diabetic and insulin therapy,” or “Diabetic and other or no therapy.” After implementing information integration for all such variables in the 50 candidate variables, Strategy B resulted in 32 variables (referred to as Set B). Details of the feature engineering performed in Strategy B and the corresponding Set B variables can be found in S3 Table.

#### Variable selection

The variable-selection stage further reduces the number of variables used in the prediction model by evaluating changes in performance from adding or removing variables. Variable selection considers each variable with respect to the other variables and is dependent on the method used to model the relationship between variables and outcome. We considered 3 variable-selection methods, including stepwise backward selection with logistic regression, lasso regularization with logistic regression, and permutation-based selection with extreme gradient boost (XGBoost). Stepwise backward selection, used in developing the baseline model, removes variables sequentially according to their strength of association with the outcome until the adjusted R^2^ reached 95% of the full model. A total of 11 variables were selected for the baseline model. To be consistent with the number of variables used in this model, we implemented stepwise backward selection to choose 11 variables. Lasso regularization, also known as the shrinkage method, selects variables by shrinking the coefficients of less-important variables from logistic regression to zero [22,23]. Existence of multicollinearity among variables was examined, and, if found, the group lasso was implemented instead [19]. The permutation-based selection is an adapted method from the Boruta algorithm [24]. First, from each of the original variables, a “shadow” variable was created by randomly permuting all the values of the original variable. Then, the prediction model (XGBoost) was run with both the original variables and their shadow variables. The variables that had higher importance in the prediction model than all the shadow variables were selected.

#### Relationship modeling

The relationship modeling stage learns the relationship between the outcome and the selected variables. We considered 2 methods, including the logistic regression method, which was implemented in the baseline model, and the machine learning method XGBoost. XGBoost is a type of gradient descent boosting that makes predictions based on a series of decision trees [25]. Tree-based modeling methods are able to naturally learn higher-order interactions and account for nonlinear relationships without predefined assumptions and thus may be more effective in capturing the potentially complex relationship between the variables and outcome.

#### Models for comparison

To understand the contribution of different methods and strategies used in developing the prediction model, we studied and compared the performance of 9 models, as illustrated in Fig 1 (Model 0 to Model 8), among which Model 1 was the replication of the current AKI model using 11 variables for clinical implementation (baseline model). From Strategy A and the resulting 20 Set A variables, Models 1, 3, and 5 implemented different variable-selection methods (backward selection, lasso regularization, and permutation selection with XGBoost) to compare the effectiveness of these variable-selection methods. Models 1 and 2 used the same selected variables but implemented different relationship-modeling methods (logistic regression and XGBoost, respectively) to test whether XGBoost could capture underlying relationships more effectively. Also, Model 1 was the reduced version of Model 0, and Model 5 was the reduced version of Model 4. The full models served as references for the corresponding reduced models, and the goal of the reduced model was to have a model using fewer variables to support more-feasible implementation in clinical care while performing almost as well as the full model. In particular, Model 0, as the full version of Model 1, was designed for providing quality assessment. In addition, Model 0 and Model 4 used the same set of variables (Set A variables) but implemented logistic regression or XGBoost as the relationship-modeling methods, respectively, and thus were also used for comparing the effectiveness of these methods in capturing the relationship between the variables and outcome. Models 6, 7, and 8 were developed using Strategy B and the resulting 32 Set B variables. Strategy B differed from Strategy A in the preprocessing stage, as Strategy B did not implement any subjective selection or transformation of variables. Models developed using Strategy B were compared against models using Strategy A to test whether availing all candidate variables to model building could produce a better model. Further, Model 6 and Model 7 differed in the variable-selection method in which lasso regularization and permutation selection were implemented, respectively. Finally, Model 7 was the full version of Model 8 as a reference for the reduced model.

In summary, a total of 6 models were developed by using Strategy A and Set A variables, and 3 models were developed by using Strategy B and Set B variables. Models 1, 2, 3, 5, 6, and 8 were reduced models in which variable selection was implemented; Models 0, 4, and 7 were full models without reduction of variables and were used as references for the corresponding reduced models.

### Statistical analyses

#### Model development and evaluation

To develop and compare the models, we repeated the following procedure 100 times (Fig 1): First, we randomly split the analytic cohort into a training set (70% of the cohort) and a test set (30% of the cohort). Second, the 9 models for predicting AKI risk were built using data from the training set only, and the corresponding selected variables were recorded. Finally, the performance of the models was assessed on the internal test set. The performance was reported by mean and 95% confidence interval (CI) from the 100 experiments.

#### Performance metrics

We used the area under the receiver operating characteristic curve (AUC, also known as the c-statistic) to measure model discrimination [26]. Calibration was assessed using the calibration slope and the reliability measure [27]. The calibration slope was estimated as the regression slope of the observed rates versus the deciles of predicted risks of AKI. The reliability measure captures the average deviations of predicted risks from observed rates for patients of varying risks, defined as the mean squared error (MSE) between the deciles of predicted risks and the observed rates, illustrated in S2 Fig. Since the reliability measure captures more granular deviations of the predicted risks from the true rates than the calibration slope and thus is a more sensitive measure of calibration, we used reliability as the primary measure to compare calibration performance of the models. Models with lower values of reliability indicate higher consistency between the predicted and observed risks and are models with better performance. We assessed the accuracy of probabilistic predictions by the Brier score, which is defined as the MSE between the observed outcome and predicted AKI risk [28]. In addition, we calculated the resolution measure, which differs from the discrimination and calibration measures; it measures how much the predicted risks of individual patients differ from the population average. The resolution measure, together with calibration measure, thus provides valuable information on the ability of the model to separate patients according to their risks for more-precise risk stratification. Resolution is calculated as the MSE between the deciles of predicted risks and the event rate of the entire cohort. Models with higher values of resolution indicate greater difference between the observed risks in predicted risk strata and the overall event rate and are models with better performance.

Among all new models with variable reduction, the best-performing model was chosen as the proposed new model to improve AKI risk prediction over the baseline model. For the baseline model and the best-performing new model, we also compared their predictive range by the difference of the observed rate between the first and 10th deciles of predicted risks through a one-sided *t* test. To further investigate the difference in predictions for individual patients made by the 2 models, using patients and their predictions in an internal test set from the experiments, we compared the mean risks predicted by the 2 models with the observed rates, for patients in the lowest and highest deciles of predicted risks identified by the model with wider predictive range. We also examined the individual patient changes in predicted risk strata between the 2 models via a shift table and compared the accuracy of risk stratification between the 2 models by calculating the observed rates for these patients.

### Sensitivity analysis

To test the effect of imputation for the variable of LVEF that had a higher missing rate than other variables, we repeated variable selection for models using LVEF, including an additional dummy variable indicating whether the value of LVEF was imputed. To test the potential predictive power of race and ethnicity variables, we repeated the procedure in Fig 1 including 5 additional race variables and 1 Hispanic ethnicity variable (See S1 Table). Further, to examine whether separate models were needed for patients with different clinical settings, using the same cohort, we assessed and compared the model performance between elective and nonelective PCIs and between PCIs that were performed or not performed the same day of the admission. Finally, to examine the performance of the model on the identification of patients with severe AKI requiring higher levels of support, we assessed the discrimination of the model for identifying patients with AKI requiring new dialysis.

#### Validation on contemporary data

To test the generalizability of the new model to contemporary practice, we also evaluated the baseline model and the best-performing new model on more contemporary data from the registry, which were not included in the main analysis. In addition to applying the models directly on the new cohort, we also considered 3 strategies that update the previously developed models for use in the new cohort. To determine whether the update is necessary and, if so, the optimal updating strategy, we split the new cohort randomly into a 70% updating and 30% validation set; the updating set was used to update the models, and the validation set was used to evaluate the performance (S3 Fig). Directly applying the previously developed models without modification was considered the baseline strategy (Strategy 1); Strategy 2 was a simple logistic recalibration method that updates the calibration intercept and slope, as is done for the current AKI model; Strategy 3 rebuilt the models using the updating set of the contemporary cohort; Strategy 4 rebuilt the models using combined data from the updating set of the contemporary cohort and the development cohort; the rebuilt model was also recalibrated via logistic calibration on the updating set. Strategies 3 and 4 were applied to explore the potential improvements of rebuilding the model with new data. The random splitting of the contemporary cohort was repeated 100 times, and the performance of the updated models by the 4 different strategies was reported by mean and 95% CI from the 100 experiments.

All analyses were developed in R (version 3.4.0) [29]. Lasso regularization with logistic regression was performed using the R package Glmnet (version 2.0–13) [30]. XGBoost was performed using the xgboost (version 0.6–4) R package [31]. Brier score, reliability, and resolution were calculated with the SpecsVerification (version 0.5–2) R package [32].

## Results

Among the 947,091 patients who underwent PCIs included in the analytic cohort, the mean (standard deviation [SD]) of their age was 64.8 (12.2) years, and 636,078 (32.8%) were women (S4 Table). A total of 69,826 (7.4%) patients developed AKI. Compared with patients without AKI, patients with AKI were 4 years older, more often female (38.9% versus 32.4%), and more often black (10.9% versus 7.7%). Patients with AKI also had worse glomerular filtration rate and were more likely to have diabetes, prior heart failure, cardiogenic shock, and cardiac arrest within 24 hours before PCI.

### Model performance comparison

For each of the 100 experiments, all models were developed using a different random 662,935 (70%) patients and evaluated in the remaining 284,126 (30%) patients.

#### Models with different variable-selection methods

**Fig 2:**
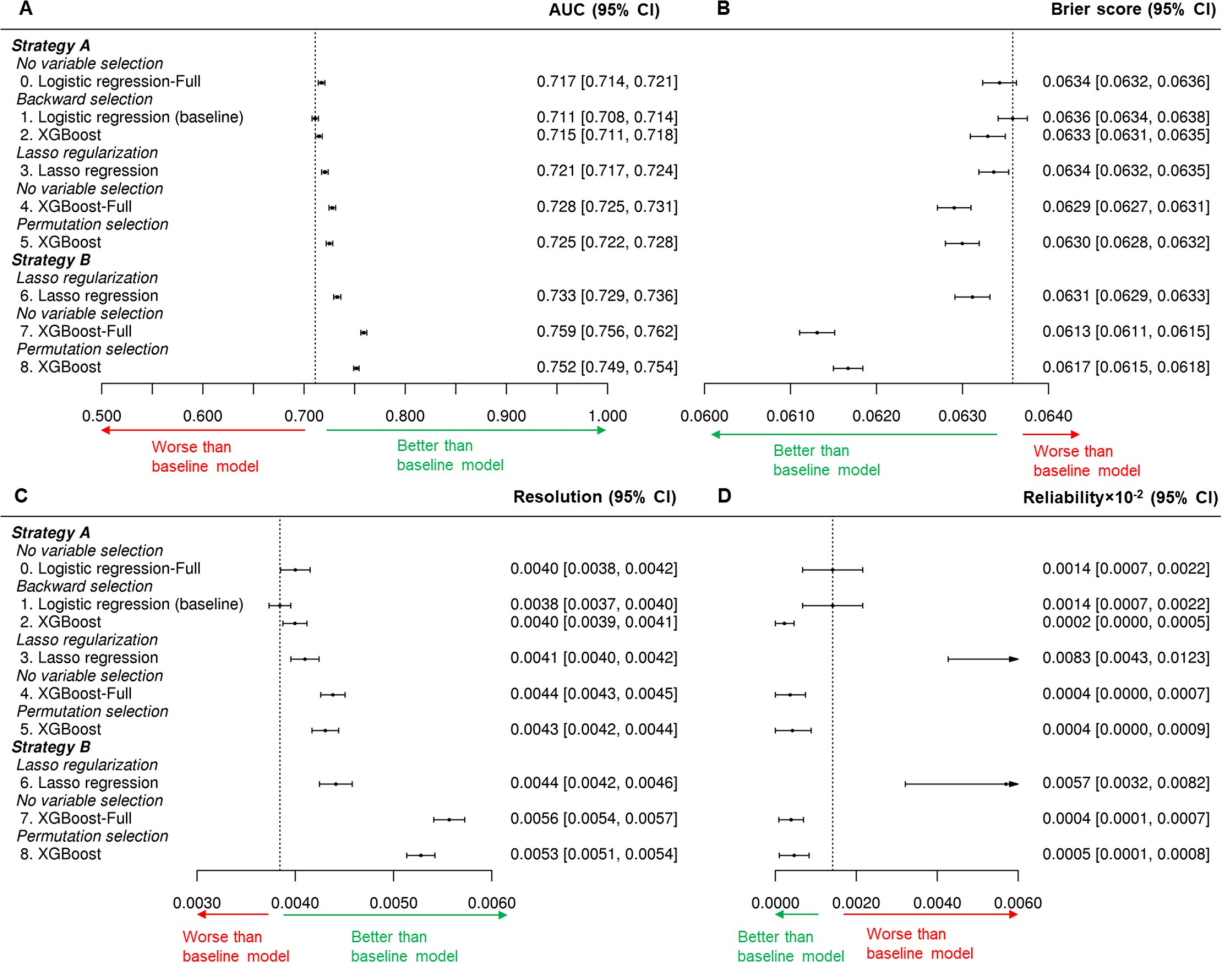
Comparison of model performance with 95% CIs for (A) the AUC, (B) Brier score, (C) resolution, and (D) reliability. AUC, area under the receiver operating characteristics curve; CI, confidence interval; XGBoost, extreme gradient boost.

Fig 2 summarizes the performance of all models developed in this study. Among Models 1, 3, and 5, which used different variable-selection methods, Model 1 had the lowest AUC (0.711; 95% CI 0.708–0.714), worst Brier score (0.0636; 95% CI 0.0634–0.0638), and lowest resolution (0.0038; 95% CI 0.0037–0.0040) and had a median level of reliability among the 3 models (0.0014 × 10^−2^; 95% CI 0.0007 × 10^−2^–0.0022 × 10^−2^), consistent with the reported performance of the baseline model. The variables selected by the different methods from 100 repeated experiments are presented in S5 Table. All variables selected in the baseline model were selected at least once by backward selection from the 100 experiments, but there were variations in selected variables among the experiments. Lasso regularization selected the same 16 variables, and permutation with XGBoost selected the same 13 variables for every iteration of the 100 experiments. Finally, Model 5, the XGBoost model using permutation selection, had the highest AUC (0.725; 95% CI 0.722–0.728), best Brier score (0.0630; 95% CI 0.0628–0.0632), highest resolution (0.0043; 95% CI, 0.0042–0.0044), and best reliability (0.0004 × 10^−2^; 95% CI 0.0000 × 10^−2^–0.0009 × 10^−2^). Of note, Model 1 had no significant difference from Model 0 (the full version of Model 1) in any performance metric; Model 5 also had no significant difference from Model 4 (the full version of Model 5) in any performance metric.

#### Models with different relationship-modeling methods

Model 0, using all Set A variables and logistic regression, had an AUC of 0.717 (95% CI 0.714–0.721), a Brier score of 0.0634 (95% CI 0.0632–0.0636), a resolution of 0.0040 (95% CI 0.0038–0.0042), and a reliability of 0.0014 × 10^−2^ (95% CI 0.0007 × 10^−2^–0.0022 × 10^−2^). Using the same variables as Model 0, Model 4, using XGBoost as the relationship-modeling method, had a significantly higher AUC (0.728; 95% CI 0.725–0.731), better Brier score (0.0629; 95% CI 0.0627–0.0631), higher resolution (0.0044; 95% CI 0.0043–0.0045), and better reliability (0.0004; 95% CI 0.0000–0.0007). Similarly, using the same variables as Model 1, Model 2, using XGBoost as the relationship-modeling method, had a slightly but not significantly higher AUC, better Brier score, higher resolution, and significantly better reliability.

#### Models with Strategy A versus Strategy B during preprocessing

Three models were developed using Strategy B and had higher AUCs than models using Strategy A. The XGBoost models using Strategy B (Models 7 and 8) additionally had a significantly better Brier score and higher resolutions than models using Strategy A. Further, from Set B variables, Model 8, which used permutation selection, achieved a significantly higher AUC, better Brier score, higher resolution, and better reliability than Model 6 did using lasso regularization, consistent with the results of comparing variable-selection methods from Set A variables. Variables selected from these 2 models can be found in S6 Table. Except for AUC, there was no significant difference between Model 8 and Model 7 (the full version of Model 8) in all performance metrics.

#### Baseline model versus best-performing new model

Among all models using variable selection (Models 2, 3, 5, 6, and 8), Model 8, the XGBoost model using 13 variables selected by permutation selection from Set B variables, had the best performance in AUC, Brier score, and resolution and was among the top in reliability. Further, compared with both the baseline model using 11 variables (Model 1) and the full version of the baseline model using 20 variables (Model 0), Model 8 had significantly better discrimination (AUC), Brier score, resolution, and reliability. The variables selected in Model 8 compared with variables used in the baseline model are presented in Table 1. The 2 models shared 9 common variables, out of which 5 had different feature engineering applied. In addition, the baseline model selected 2 other variables, whereas Model 8 selected another 4 variables.

**Table 1:** Comparison of variables used in the baseline model (Model 1) and the XGBoost model (Model 8).

	Baseline model (*N* = 11)	XGBoost model (Model 8) (*N* = 13)
Same variables	Age	Age
	Prior heart failure	Prior heart failure
	Cardiogenic shock within 24 hours (no versus yes)	Cardiogenic shock within 24 hours (no versus yes)
	Cardiac arrest within 24 hours (no versus yes)	Cardiac arrest within 24 hours (no versus yes)
Same variables with different feature engineering	Diabetes mellitus (no versus yes)	Diabetes mellitus composite (no versus yes, insulin versus yes, others)
	CAD presentation (stable CAD versus non-STEMI or unstable angina versus STEMI)	CAD presentation composite (non-STEMI versus others)
	Heart failure within 2 weeks (no versus yes)	Heart failure within 2 weeks composite (no versus yes, NYHA class IV versus yes, others)
	Preprocedure GFR (normal versus mild versus moderate versus severe)	Preprocedure GFR
	Anemia (preprocedure hemoglobin < 10) (no versus yes)	Preprocedure hemoglobin
Different variables	Cerebrovascular disease (no versus yes)	
	IABP at the start of procedure (no versus yes)	
		Admission source (emergency department versus others)
		Body mass index
		PCI status (elective versus emergency versus others)
		Pre-PCI left ventricular ejection fraction

Details of feature engineering performed in Models 1 and 8 can be found in S2 Table and S3 Table, respectively.

Abbreviations: CAD, coronary artery disease; GFR, glomerular filtration rate; IABP, intra-aortic balloon pump; NYHA, New York Heart Association; PCI, percutaneous coronary intervention; STEMI, ST elevation myocardial infarction; XGBoost, extreme gradient boost.

**Fig 3:**
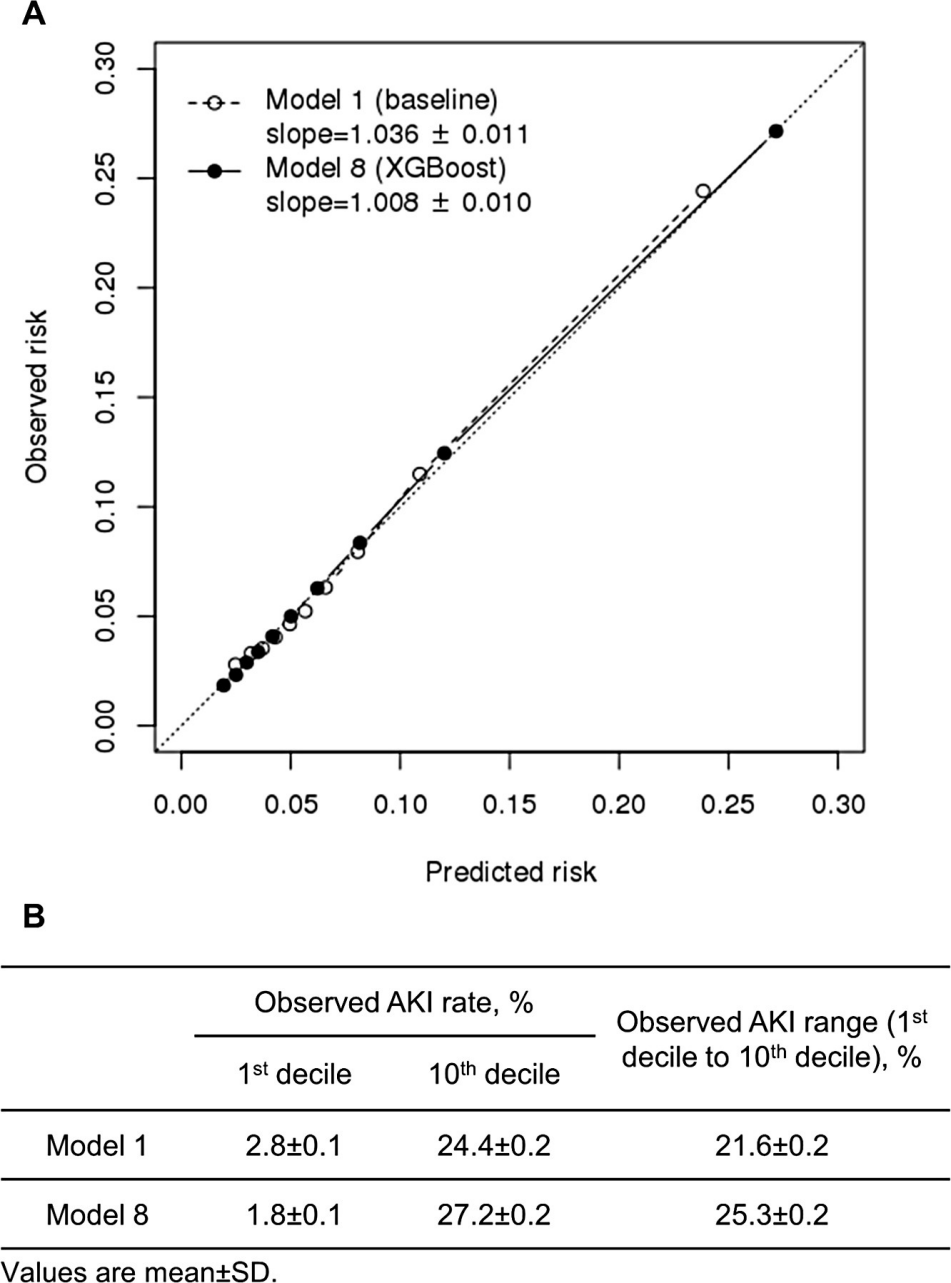
Comparison of the baseline model (Model 1) and the XGBoost model (Model 8) in (A) calibration and (B) predictive range. AKI, acute kidney injury; SD, standard deviation; XGBoost, extreme gradient boost.

The observed AKI rate in deciles of risk predicted by Model 1 (the baseline model) and Model 8 are plotted in Fig 3A. Model 8 showed better calibration than Model 1 in the calibration slope (1.008 versus 1.036, *p* < 0.001) and in the calibration intercept (−0.001 versus −0.003, *p* < 0.001). Further, shown in Fig 3B, Model 8 had a wider predictive range than Model 1 had (25.3% versus 21.6%, *p* < 0.001) and more accurate risk prediction for lowest and highest deciles of patients than Model 1 (S7 Table). Table 2 shows that patients stratified by both the baseline Model 1 and the newly developed Model 8 had consistent observed rates with their risk strata. However, for the 103,556 (36.4%) patients (in off-diagonal entries of Table 2) that had different risk strata predicted by Model 1 and Model 8, Model 8 was more accurate in estimating their risks compared with their actual observed rate. More specifically, Model 8 accurately reclassified a total of 42,167 (14.8%) patients whose risks were underestimated by Model 1 and a total of 61,388 (21.6%) patients whose risks were overestimated by Model 1. The between-model difference in risk stratification was most prominent in small subgroups of 124 (0.04%) patients and 103 (0.04%) patients with predicted risks of 25%–50% and higher than 50% by Model 8, respectively. They were estimated by Model 1 to have a risk less than 5%, whereas their observed rates were in fact 39.5% and 71.8%.

**Table 2:** Shift table of predicted risks from the baseline model (Model 1) and the XGBoost model (Model 8).

	Model 1 predicted risk					
	<5%	5%–10%	10%–25%	25%–50%	>50%	All
Model 8 predicted risk	observed rate (No. patients)	observed rate (No. patients)	observed rate (No. patients)	observed rate (No. patients)	observed rate (No. patients)	observed rate (No. patients)
<5%	2.8% (108,015)	3.5% (46,808)	4.2% (1,537)	0% (12)	NA (0)	3.0% (156,372)
5%–10%	6.2% (18,551)	7.1% (45,115)	8.9% (10,448)	6.1% (82)	NA (1)	7.1% (74,197)
10%–25%	13.2% (2,241)	13.0% (15,185)	16.5% (22,291)	24.2% (1,925)	21.7% (23)	15.4% (41,665)
25%–50%	39.5% (124)	31.1% (412)	31.1% (4,466)	36.7% (4,427)	43.4% (553)	34.3% (9,982)
>50%	71.8% (103)	76.3% (93)	53.8% (171)	51.8% (821)	58.6% (722)	56.8% (1,910)
All	3.6% (129,034)	6.5% (107,613)	15.8% (38,913)	34.6% (7,267)	51.4% (1,299)	7.4% (284,126)

The cells show the observed AKI rate for patients with different predicted risk strata between Model 1 and Model 8. The numbers in parentheses are the total number of patients in the cell from which the observed rate was evaluated. The observed AKI rate was not calculated and was denoted as NA if the number of patients was fewer than 10. Shaded entries are subgroups of patients who had same predicted risk strata by Model 1 and Model 8.

Abbreviations: AKI, acute kidney injury; XGBoost, extreme gradient boost.

### Sensitivity analyses

The dummy variable indicating imputation for LVEF was not selected by permutation selection in Model 8. Adding race and ethnicity variables to models did not significantly improve the model performance, and these variables were also not selected in Model 8. There was no significant difference in the performance of Model 8 when performed independently for elective versus nonelective PCI patients. No difference in performance was found for patients with same-day versus not-same-day PCI from their admission. Finally, Model 8 performed better for identifying AKI requiring new dialysis than identifying AKI, with an AUC of 0.911 (95% CI 0.903–0.918).

#### Temporal validation

Baseline characteristics and outcome for patients included in the development cohort for building the models and in the more contemporary cohort for temporal validation were summarized in S8 Table. Compared with the development cohort, patients in the contemporary cohort had a higher AKI rate and were 1 year older, more often African Americans, and more likely to have prior heart failure, diabetes, cardiogenic shock, and cardiac arrest within 24 hours of PCI. When directly applied on the contemporary cohort without updating (Strategy 1), the best-performing new model (Model 8) outperformed the baseline model (Model 1) in AUC (0.785 versus 0.753), Brier score (0.0610 versus 0.0627), calibration slope (1.003 versus 1.062), and resolution (0.0073 versus 0.0057) (S4 Fig, S9 Table). Model 8 also had a wider predictive range than Model 1 had (29.4% versus 26.2%) on the contemporary cohort. Minimal improvement was seen by implementing updating strategies (Strategy 2–4), except for significant improvement in reliability in both Model 1 and Model 8 via recalibration (Strategy 2).

## Discussion

We found that a model for predicting AKI risk after PCI, derived from data-driven approaches of building prediction models and with the use of machine learning methods, had small but statistically significant improvement in performance over the current ACC NCDR CathPCI Registry AKI model, which was built using hierarchical logistic regression [7]. The machine learning model, compared with the current AKI model, had a wider predictive range, was more accurate in stratifying risk of AKI for patients, and, for a small subset, resulted in marked changes in their estimates, which has important clinical implications. The improvement in performance of the proposed machine learning model over the current AKI model was a result of employing all available variables to modeling, using permutation test for variable selection and implementing XGBoost to model the relationship between variables and outcome. Further, the best machine learning model only entailed the use of 2 more variables than the current AKI model, posing minimal additional burden on data extraction and processing.

Traditional strategies of developing prediction models in medicine have contributed much to current quality improvement and decision support. However, they have some limitations that could lead to missing important predictors and relationships [1,2,7,33]. The proposed XGBoost model was developed as an effort to overcome the limitations presented by traditional strategies of building prediction models. Through developing and comparing a total of 9 models, we derived a prediction model for AKI risk after PCI by optimizing strategies or methods in various stages of model development, and we were able to understand how the improvement in the new model was achieved. First, we found that models implementing permutation selection with XGBoost performed better than those using stepwise selection and lasso regularization with logistic regression. This demonstrates the improvement due to the machine learning–based variable-selection method. Second, using the same variables, using XGBoost to model the relationship performed better than the baseline model using logistic regression, showing the superior modeling capacity of machine learning methods to learn complex relationships between the variables and outcome. Finally, models having access to all available candidate variables in their original forms performed better than those constrained to a reduced set of variables that had been selected in the development of the baseline model. This is evidence that there was loss in model performance due to investigator-driven preprocessing of variables. Moreover, the significant improvement by XGBoost models using Strategy B over those using Strategy A further showed that presenting all available information is important for machine learning models to reach their maximum performance potential. Finally, the full models performed better than their reduced versions. Thus, while using models with fewer variables may be worthwhile for more feasible prospective implementation, the more complete models may be better for retrospectively generating quality assessment reports.

Although statistically significant, the absolute improvement in population-based performance measures may not seem large. However, our results on more accurate risk stratification of individual patients further showed that a small minority of patients had marked changes in their risk estimates. Improvement in overall performance measures, which are the key standard metrics in comparing models, do not present the entire picture of the potential benefits of a machine learning–based model and may be insensitive to reflect improvement for small subgroups of patients. The machine learning model also had a significantly wider predictive range, which extends the limit of the current AKI model in identifying patients with the lowest and highest risks. In addition, the machine learning model was shown to provide a more accurate estimation of risks for patients with extremely low and high risks, whereas the current AKI model over- or underestimated their risks. Thus, the machine learning model may be particularly useful for patients at the extreme ends of the risk spectrum. In fact, for all 103,555 (36.4%) patients who had risk strata predicted differently by the 2 models, the machine learning model demonstrated higher consistency between predicted risk strata and true event rates.

The improved accuracy of risk stratification by the machine learning model may have important implications for the management of patients undergoing PCI, especially for identifying patients at risk for AKI who may benefit from more intensive risk-mitigation strategies. For example, the 2,468 (0.9%) patients estimated by the current AKI model to have a risk of AKI less than 5% in fact could more accurately be classified as having at least a 10% risk of developing AKI from the machine learning model. Marked underestimation by the current AKI model was also seen in a very small subgroup of 227 (0.1%) patients predicted to be under 5% risk but who could more accurately be classified as having at least a 25% observed risk. On the other hand, the low-risk subgroup of 1,537 (0.5%) patients classified by the current AKI model to have an elevated risk of 10%–25% were more accurately estimated by the machine learning model to have a risk less than 5%. Correctly identifying the risk of patients for AKI can be an important guide in risk-mitigation interventions, and given the small number of patients with marked change in risk estimates, the improved abilities of machine-learning models should be explored to see if they could better improve safety over the current risk models.

The evolution of greater computational capacities of electronic health records (EHRs) offers the potential for calculating risk estimates at the point of care using machine learning–based models. For example, the eHospital launched by Cleveland Clinic trained tree-based machine learning models on clinical data from EHR and deployed the trained model as a web service that can take real-time data from a patient in the intensive care unit to provide timely prediction of the probability of this patient needing vasopressor therapy [34]. Similarly, the machine learning model we developed may provide better risk adjustment in AKI quality assessment and comparative benchmarking to support quality improvement, as is done in clinical registry programs such as the NCDR.

Despite the statistical advantages and potential clinical implications of machine learning demonstrated in these analyses, there are certain barriers to its larger adoption in medicine. First, logistic regression models are more familiar to clinicians than machine learning techniques, and the linear modeling permits the generation of coefficients, which help clinicians understand the strength of the relationship between patient characteristics and outcome. In spite of demonstrated improvement in performance, the interpretation of the proposed machine learning model and the association between predictors and outcome is difficult, since the model makes predictions based on multiple decision trees. There are measures for quantifying the strength of relationships in machine learning models, but they are more complicated. Whether clinicians will accept the results of a machine learning model—and whether the more challenging interpretability is an impediment to adoption—will need to be studied. Second, there is experience with the operationalization of regression-based models in routine clinical care, and they have been shown to influence outcomes [35–37]. Whether machine learning techniques could be similarly used to improve care is an important area for future research. Finally, in low-resource environments, logistic regression–based models can still be implemented using simple integer scores.

Our study has several limitations. First, relationships in data may change over time because of changing coding patterns and changing populations, and the new model may not perform as well when applied to newer data [38]. Although we have tested our developed model on more contemporary NCDR CathPCI data, we anticipate that the model will need to be continually updated, as is done with the current AKI model. Second, the new model, although with improved performance over the current AKI model, remains a static model. With the availability of time series data of changing patient parameters with integrated EHRs, real-time dynamic prediction at different time points to facilitate decision-making throughout the patient’s entire hospitalization is possible and is an avenue for future work [39]. Third, the potential of these models ultimately resides in point-of-care application using readily available data. For this study, we used registry data and not EHR data. We did so because we were comparing against the NCDR model, which is the current gold standard for AKI risk estimation. Future studies may take the next step to leverage data available in the EHRs. Challenges of integrating the prediction model to EHRs include the availability and accessibility of the predictors from EHRs at the point of decision. Finally, this study does not address implementation. Medicine has yet to fully integrate risk calculations into clinical care, and even more-complete adoption of existing models could improve care, particularly if they are accompanied by management recommendations. This paper was restricted to addressing the question of whether the current AKI risk model could be improved based on the same data with which it was derived. Although we have focused on predicting AKI risks, which has important clinical significance, the study serves as a case study to understand the mechanisms and conditions under which machine learning techniques improve prediction over traditional statistical techniques.

In conclusion, this study developed a machine learning–based model to predict AKI risk after PCI through a data-driven approach. Using machine learning techniques that could use all available information for variable selection and relationship modeling, we demonstrated an improved prediction for AKI risk after PCI compared with the current model based on regression techniques. With the prevalence of advanced computing and integrated EHRs at the bedside, predictive models developed with these techniques have the potential to be incorporated into routine patient care as well as to support quality improvement.

## Supporting information

S1 Checklist TRIPOD Checklist. TRIPOD, transparent reporting of a multivariable prediction model for individual prognosis or diagnosis.
(DOCX)

S1 Table Candidate variables (*N* = 50). Models were developed both without and with race and ethnicity variables.
(DOCX)

S2 Table Set A variables (*N* = 20) and feature engineering in strategy A during preprocessing. (DOCX)

S3 Table Set B variables (*N* = 32) and feature engineering in strategy B during preprocessing. (DOCX)

S4 Table Patient characteristics for the cohort used in developing the models. (DOCX)

S5 Table Variable-selection results from set A variables (*N* = 20) from 100 experiments. (DOCX)

S6 Table Variable-selection results from set B variables (*N* = 32) from 100 experiments. (DOCX)

S7 Table Prediction accuracy of the baseline model (Model 1) and XGBoost model (Model 8) for low- and high-risk subgroups of patients. XGBoost, extreme gradient boost.
(DOCX)

S8 Table Patient characteristics for the development cohort and the contemporary cohort. (DOCX)

S9 Table Summary of data used for building model or recalibration and performance of updated model from Model 1 (baseline model) and the Model 8 (XGBoost model) for 4 different strategies. XGBoost, extreme gradient boost.
(DOCX)

S1 Fig Flowchart of study participants for (A) the development cohort for the main analysis and (B) the contemporary cohort for temporal validation. PCI, percutaneous coronary intervention.
(DOCX)

S2 Fig Illustration of reliability and resolution metrics. The x-axes of the points are the deciles of predicted risks, and the y-axes of the points are the observed event rate of the patients in each decile.
(DOCX)

S3 Fig Analysis flow for evaluating previously developed prediction models on a more contemporary cohort. (DOCX)

S4 Fig Performance comparison with 95% CIs of the updated models via 4 strategies in the AUC, reliability, resolution, and Brier score from Model 1 (baseline model) and Model 8 (XGBoost model). AUC, area under the receiver operating characteristics curve; CI, confidence interval; XGBoost, extreme gradient boost.
(DOCX)

## Abbreviations

ACC: American College of Cardiology
AKI: acute kidney injury
AUC: area under the receiver operating characteristics curve
CAD: coronary artery disease
CI: confidence interval
EHR: electronic health record
LVEF: left ventricular ejection fraction
MSE: mean squared error
NCDR: National Cardiovascular Data Registry
NYHA: New York Heart Association
PCI: percutaneous coronary intervention
SD: standard deviation
STEMI: ST elevation myocardial infarction
TRIPOD: transparent reporting of a multivariable prediction model for individual prognosis or diagnosis
XGBoost: extreme gradient boost
