# Supplementary material for: Enhancing the prediction of acute kidney injury risk after percutaneous coronary intervention using machine learning techniques: A retrospective cohort study
Source: PLoS Med. 2018 Nov 27;15(11):e1002703. doi: 10.1371/journal.pmed.1002703 (PMC6258473; doi:10.1371/journal.pmed.1002703)
Supplement: S1 Table — Models were developed both without and with race and ethnicity variables. (DOCX) [file pmed.1002703.s002.docx]

| **Name** | **Description** | **CathPCI Seq.#** |
| --- | --- | --- |
| Age | Patient age | 2050 |
| Sex | Patient’s sex at birth | 2060 |
| Admit Source | Source of admission (emergency department/transfer in from another acute care facility/other) | 3010 |
| Current/Recent Smoker | Smoked cigarettes anytime during the year prior to arrival (no/yes) | 4000 |
| Hypertension | Has a current diagnosis of hypertension (no/yes) | 4005 |
| Dyslipidemia | Has a history of dyslipidemia diagnosed and/or treated (no/yes) | 4010 |
| Family History of Premature CAD | Has a family history of premature coronary artery disease (no/yes) | 4015 |
| Prior MI | Had at least 1 documented previous myocardial infarction (no/yes) | 4020 |
| Prior Heart Failure | Previous history of heart failure (no/yes) | 4025 |
| Prior Valve Surgery/Procedure | Had a previous surgical replacement and/or repair of a cardiac valve (no/yes) | 4030 |
| Prior PCI | Had a previous percutaneous coronary intervention (no/yes) | 4035 |
| Most Recent PCI Date | Date of the most recent PCI | 4040 |
| Prior CABG | Had a previous coronary artery bypass grafting (no/yes) | 4045 |
| Most Recent CABG Date | Date of the most recent CABG | 4050 |
| Height | Patient’s height in centimeters | 4055 |
| Weight | Patient’s weight in kilograms | 4060 |
| Cerebrovascular Disease | Has a history of cerebrovascular disease (no/yes) | 4070 |
| Peripheral Arterial Disease | Has a history of peripheral arterial disease (PAD) (no/yes) | 4075 |
| Chronic Lung Disease | Has a history of chronic lung disease (no/yes) | 4080 |
| Diabetes Mellitus | Has a history of diabetes mellitus (no/yes) | 4085 |
| Diabetes Therapy | The most aggressive diabetes therapy the patient presented with (none/diet/oral/insulin/other) | 4090 |
| CAD Presentation | Patient’s coronary artery disease presentation (no symptoms, no angina/symptoms unlikely to be ischemic/stable angina/unstable angina/non-STEMI/STEMI) | 5000 |
| Thrombolytics | Received thrombolytic therapy (no/yes) | 5010 |
| Anginal Classification w/in 2 Weeks | Patient’s anginal classification or symptom status within the past 2 weeks (no symptoms, no angina/CCS I/CCS II/CCS III/CCS IV) | 5020 |
| Anti-Anginal Medication w/in 2 Weeks | Has taken or has been prescribed anti-anginal medication within the past 2 weeks (no/yes) | 5025 |
| Beta Blockers | Has taken or has been prescribed a beta blocker to treat anginal symptoms (no/yes) | 5026 |
| Calcium Channel Blockers | Has taken or has been prescribed a calcium channel blocker to treat anginal symptoms (no/yes) | 5027 |
| Long Acting Nitrates | Has taken or has been prescribed long acting nitrates to treat anginal symptoms (no/yes) | 5028 |
| Ranolazine | Has taken or has been prescribed ranolazine to treat anginal symptoms (no/yes) | 5029 |
| Other Anti-Anginal Agent | Has taken or has been prescribed any other anti-anginal medications to treat anginal symptoms (no/yes) | 5030 |
| Heart Failure w/in 2 Weeks | Has been in a state of heart failure within the past 2 weeks (no/yes) | 5040 |
| NYHA Class w/in 2 Weeks | Patient’s worst dyspnea or functional class (Class I/Class II/Class III/Class IV) | 5045 |
| Cardiomyopathy or Left Ventricular Systolic Dysfunction | A reason for the cath lab visit is evaluation of cardiomyopathy and/or left ventricular systolic dysfunction (no/yes) | 5050 |
| Cardiogenic Shock w/in 24 Hours | Has been in a state of cardiogenic shock within 24 hrs of procedure (no/yes) | 5060 |
| Cardiac Arrest w/in 24 Hours | Had an episode of cardiac arrest within 24 hours of procedure (no/yes) | 5065 |
| Stress or Imaging Studies | If an exercise stress test, stress echocardiogram, stress testing with SPECT MPI, stress testing with CMR, cardiac CTA or coronary calcium scoring was performed (no/yes) | 5100 |
| IABP | If the patient required the use of an intra-aortic balloon pump (no/yes) | 5330 |
| IABP Timing | (in place at start of procedure/inserted during procedure and prior to PCI/inserted after PCI has begun) | 5335 |
| Other Mechanical Ventricular Support | If the patient required the use of other mechanical ventricular support (no/yes) | 5340 |
| Other Mechanical Ventricular Support Timing | When the other mechanical ventricular support was placed (in place at start of procedure/inserted during procedure and prior to PCI/inserted after PCI has begun) | 5345 |
| PCI Status | The status of PCI (elective/urgent/emergency/salvage) | 7020 |
| Pre-PCI Ventricular Ejection Fraction | Pre-PCI Left Ventricular Ejection Fraction | 7025 |
| Pre-procedure Creatinine | Patient’s most recent creatinine level in mg/dL | 7316 |
| Pre-Procedure Hemoglobin | The most recent hemoglobin level in g/dL | 7320 |
| Race and Ethnicity variables | | |
| Race-White | If the patient is White as determined by the patient/family (no/yes) | 2070 |
| Race-Black or African American | If the patient is Black or African American as determined by the patient/family (no/yes) | 2071 |
| Race-Asian | If the patient is Asian as determined by the patient/family (no/yes) | 2072 |
| Race-American Indian or Alaskan Native | If the patient is American Indian or Alaskan Native as determined by the patient/family (no/yes) | 2073 |
| Race-Native Hawaiian or Pacific Islander | If the patient is Native Hawaiian or Pacific Islander as determined by the patient/family (no/yes) | 2074 |
| Hispanic or Latino Ethnicity | If the patient is of Hispanic or Latino ethnicity as determined by the patient/family (no/yes) | 2076 |

CAD indicates coronary artery disease; MI, myocardial infarction; PCI, percutaneous coronary intervention; CABG, coronary artery bypass grafting; CCS, Canadian Cardiovascular Society Classification System; NYHA, New York Heart Association; IABP, intra-aortic balloon pump; STEMI, ST-elevation myocardial infarction.
