# Supplementary material for: Enhancing the prediction of acute kidney injury risk after percutaneous coronary intervention using machine learning techniques: A retrospective cohort study
Source: PLoS Med. 2018 Nov 27;15(11):e1002703. doi: 10.1371/journal.pmed.1002703 (PMC6258473; doi:10.1371/journal.pmed.1002703)
Supplement: S2 Table — (DOCX) [file pmed.1002703.s003.docx]

| **Variable name** | **Description of feature engineering, if any** |
| --- | --- |
| Age |  |
| Sex |  |
| Transfer-in status |  |
| Hypertension |  |
| Prior PCI |  |
| Prior MI |  |
| Prior heart failure |  |
| Prior CABG |  |
| Body mass index | BMI calculated using “Height” and “Weight”: BMI=Weight in kilograms/(height in meters)^2^ |
| Cerebrovascular disease |  |
| Peripheral arterial disease |  |
| Chronic lung disease |  |
| Diabetes mellitus |  |
| CAD presentation | Combine categories into a variable that takes values of non-ACS/ non-STEMI or unstable angina/STEMI) |
| Heart failure w/in 24 hours |  |
| Cardiogenic shock w/in 24 hours |  |
| Cardiac arrest w/in 24 hours |  |
| IABP at the start of procedure | If the patient required the use of an intra-aortic balloon pump at start of procedure (no/yes) |
| Pre-procedure GFR levels | Calculated using “Age,” “Pre-procedure Creatinine,” “Race-Black or African American,” and “Sex.” Further categorized into normal (>60)/mild (45-60)/moderate (30-45)/severe (<30 mL/min) |
| Anemia | Pre-procedure hemoglobin<10 (no/yes) |

CAD indicates coronary artery disease; MI, myocardial infarction; PCI, percutaneous coronary intervention; CABG, coronary artery bypass grafting; ACS, acute coronary syndrome; IABP, intra-aortic balloon pump; STEMI, ST-elevation myocardial infarction; GFR, glomerular filtration rate.
