# Supplementary material for: Enhancing the prediction of acute kidney injury risk after percutaneous coronary intervention using machine learning techniques: A retrospective cohort study
Source: PLoS Med. 2018 Nov 27;15(11):e1002703. doi: 10.1371/journal.pmed.1002703 (PMC6258473; doi:10.1371/journal.pmed.1002703)
Supplement: S3 Table — (DOCX) [file pmed.1002703.s004.docx]

| **Variable name** | **Description of feature engineering, if any** |
| --- | --- |
| Age |  |
| Sex |  |
| Admit source |  |
| Current/Recent smoker |  |
| Hypertension |  |
| Dyslipidemia |  |
| Family history of premature CAD |  |
| Prior MI |  |
| Prior heart failure |  |
| Prior valve surgery/procedure |  |
| Prior PCI composite | Combine “Prior PCI” and “Most Recent PCI Date” into a composite variable that takes values of no/yes, within last year/yes, longer than 1 year ago |
| Prior CABG composite | Combine “Prior CABG” and “Most Recent CABG Date” into a composite variable that takes values of no/yes-within last year/yes-1-5 years/yes-longer than 5 years ago |
| Body mass index | BMI calculated using “Height” and “Weight”: BMI=Weight in kilograms/(height in meters)^2^ |
| Cerebrovascular disease |  |
| Peripheral arterial disease |  |
| Chronic lung disease |  |
| Diabetes mellitus composite | Combine “Diabetes Mellitus” and “Diabetes Therapy” into a composite variable that takes value of no/yes-oral/yes-insulin/yes-diet or yes-other or yes-none |
| CAD presentation composite | Combine “CAD Presentation” and “Thrombolytics” into a composite variable that takes values of no symptom or no angina or symptom unlikely to be ischemic/stable angina/unstable angina/non-STEMI/STEMI and received thrombolytics/STEMI and not received thrombolytics |
| Anginal classification w/in 2 weeks |  |
| Anti-anginal mediation w/in 2 weeks composite | Combine “Anti-Anginal Medication w/in 2 Weeks,” “Beta Blockers,” “Calcium Channel Blockers,” “Long Acting Nitrates,” “Ranolazine,” and “Other Anti-Anginal Agent” into a composite variable that takes values of no/yes, taking 1 medication/yes, taking more than 1 medication |
| Beta blocker |  |
| Heart failure w/in 2 weeks composite | Combine “Heart Failure w/in 2 Weeks composite” and “NYHA Class w/in 2 Weeks” into a composite variable that takes values of no/yes-Class I/yes-Class II/yes-Class III/yes-Class IV |
| Cardiomyopathy or left ventricular systolic dysfunction |  |
| Cardiogenic shock w/in 24 hours |  |
| Cardiac arrest w/in 24 hours |  |
| Stress or imaging studies |  |
| IABP at the start of procedure | If the patient required the use of an intra-aortic balloon pump at start of procedure (no/yes) |
| Other mechanical ventricular support at the start of procedure | If the patient required the use of other mechanical ventricular support at start of procedure (no/yes) |
| PCI status |  |
| Pre-PCI ventricular ejection fraction |  |
| Pre-procedure GFR | Calculated using “Age,” “Pre-procedure Creatinine,” “Race-Black or African American,” and “Sex” |
| Pre-procedure hemoglobin |  |

CAD indicates coronary artery disease; MI, myocardial infarction; PCI, percutaneous coronary intervention; CABG, coronary artery bypass grafting; NYHA, New York Heart Association; IABP, intra-aortic balloon pump; STEMI, ST-elevation myocardial infarction; GFR, glomerular filtration rate.
