# Supplementary material for: Enhancing the prediction of acute kidney injury risk after percutaneous coronary intervention using machine learning techniques: A retrospective cohort study
Source: PLoS Med. 2018 Nov 27;15(11):e1002703. doi: 10.1371/journal.pmed.1002703 (PMC6258473; doi:10.1371/journal.pmed.1002703)
Supplement: S4 Table — (DOCX) [file pmed.1002703.s005.docx]

| **Characteristic**  **N (%)** | **Total Cohort**  **947,091 (100.0)** | **AKI**  **69,826 (7.4)** | **No AKI**  **877,265 (92.6)** |
| --- | --- | --- | --- |
| No. AKI, % | 69,826 (7.4) | 69,826 (100.0) | 0 (0) |
| Age, y | 64.8 ± 12.2 | 68.2 ± 12.4 | 64.6 ± 12.1 |
| Female, n (%) | 636,078 (32.8) | 27,189 (38.9) | 283,824 (32.4) |
| Race, n (%) |  |  |  |
| White | 108,624 (88.5) | 59,610 (85.4) | 778,857 (88.8) |
| Black/African American | 74,820 (7.9) | 7,590 (10.9) | 67,230 (7.7) |
| Admission source, n (%) |  |  |  |
| Emergency department | 371,959 (39.3) | 34,053 (48.8) | 337,906 (38.5) |
| Transfer from acute care facility | 174,032 (18.4) | 17,587 (25.2) | 156,445 (17.8) |
| Body mass index, kg/m^2^ | 30.1 ± 11.8 | 30.5 ± 14.8 | 30.0 ± 11.5 |
| Baseline GFR, ml/min/1.73m^2^ | 76.5 ± 29.3 | 72.2 ± 60.4 | 76.8 ± 25.3 |
| GFR level, n (%) |  |  |  |
| Normal >=60 | 695,959 (73.5) | 39,160 (56.1) | 656,799 (74.9) |
| Mild GFR 45 to 60 | 159,691 (16.9) | 14,094 (20.2) | 145,597 (16.6) |
| Moderate GFR 30 to 45 | 71,091 (7.5) | 10,532 (15.1) | 60,559 (6.9) |
| Severe GFR <30 | 20,189 (2.1) | 5,879 (8.4) | 14,310 (1.6) |
| Anemia, n (%) | 33,988 (3.6) | 7,217 (10.3) | 26,771 (3.1) |
| Hypertension, n (%) | 774,519 (81.8) | 60,492 (86.6) | 714,027 (81.4) |
| Prior MI, n (%) | 282,352 (29.8) | 22,643 (32.4) | 259,709 (29.6) |
| Prior heart failure, n (%) | 110,003 (11.6) | 15,755 (22.6) | 94,248 (10.7) |
| Prior PCI, n (%) | 376,248 (39.7) | 25,528 (36.6) | 350,720 (40.0) |
| Prior CABG, n (%) | 176,136 (18.6) | 14,927 (21.4) | 161,209 (18.4) |
| Cerebrovascular disease, n (%) | 115,924 (12.2) | 12,905 (18.5) | 103,019 (11.7) |
| Peripheral arterial disease, n (%) | 116,020 (12.3) | 12,801 (18.3) | 103,219 (11.8) |
| Chronic lung disease, n (%) | 144,177 (15.2) | 14,204 (20.3) | 129,973 (14.8) |
| Diabetes mellitus, n (%) | 339,226 (35.8) | 34,392 (49.3) | 304,834 (34.7) |
| CAD presentation, n (%) |  |  |  |
| No symptom, no angina | 83,317 (8.8) | 4,943 (7.1) | 78,374 (8.9) |
| Symptom unlikely to be ischemic | 27,698 (2.9) | 1,820 (2.6) | 25,878 (2.9) |
| Stable angina | 156,613 (16.5) | 6,431 (9.2) | 150,182 (17.1) |
| Unstable angina | 342,774 (36.2) | 20,091 (28.8) | 322,683 (36.8) |
| Non-STEMI | 181,890 (19.2) | 19,317 (27.7) | 162,573 (18.5) |
| STEMI or equivalent | 154,525 (16.3) | 17,206 (24.6) | 137,319 (15.7) |
| IABP before procedure, n (%) | 1,944 (0.2) | 697 (1.0) | 1,247 (0.1) |
| Heart failure within 2 weeks, n (%) | 95,373 (10.1) | 17,229 (24.7) | 78,144 (8.9) |
| Cardiogenic shock within 24 hours, n (%) | 17,219 (1.8) | 5,750 (8.2) | 11,469 (1.3) |
| Cardiac arrest within 24 hours, n (%) | 17,250 (1.8) | 3,966 (5.7) | 13,284 (1.5) |

Values are mean ± SD except as noted. AKI indicates acute kidney injury; GFR, glomerular filtration rate; CABG, coronary artery bypass grafting; MI, myocardial infarction; PCI, percutaneous coronary intervention; CAD, coronary artery disease; STEMI, ST-elevation myocardial infarction.
