# Supplementary material for: Enhancing the prediction of acute kidney injury risk after percutaneous coronary intervention using machine learning techniques: A retrospective cohort study
Source: PLoS Med. 2018 Nov 27;15(11):e1002703. doi: 10.1371/journal.pmed.1002703 (PMC6258473; doi:10.1371/journal.pmed.1002703)
Supplement: S5 Table — (DOCX) [file pmed.1002703.s006.docx]

|  | **Variable selection method** | | |
| --- | --- | --- | --- |
| **Variable** | **Backward selection with logistic regression (No. times selected in top 11)** | **Lasso regularization with logistic regression (No. times selected)** | **Permutation selection with XGBoost (No. times selected)** |
| Age | 100 | 100 | 100 |
| Sex | 0 | 0 | 0 |
| Transfer-in status | 10 | 100 | 100 |
| Hypertension | 0 | 100 | 0 |
| Prior PCI | 0 | 100 | 0 |
| Prior MI | 0 | 0 | 0 |
| Prior heart failure | 100 | 100 | 100 |
| Prior CABG | 0 | 0 | 0 |
| Body mass index | 0 | 100 | 100 |
| Cerebrovascular disease | 96 | 100 | 100 |
| Peripheral arterial disease | 81 | 0 | 100 |
| Chronic lung disease | 0 | 100 | 0 |
| Diabetes mellitus | 100 | 100 | 100 |
| CAD presentation (non-ACS/non-STEMI or unstable angina/STEMI) | 100 | 100 | 100 |
| Heart failure w/in 2 weeks | 100 | 100 | 100 |
| Cardiogenic shock w/in 24 hours | 100 | 100 | 100 |
| Cardiac arrest w/in 24 hours | 100 | 100 | 100 |
| IABP at the start of procedure | 13 | 100 | 0 |
| Pre-procedure GFR (normal/mild/moderate/severe) | 100 | 100 | 100 |
| Anemia (pre-procedure hemoglobin <10) | 100 | 100 | 100 |

CAD indicates coronary artery disease; CABG, coronary artery bypass grafting; MI, myocardial infarction; STEMI, ST-elevation myocardial infarction; IABP, intra-aortic balloon pump; ACS, acute coronary syndrome; GFR, glomerular filtration rate.
