# Supplementary material for: Enhancing the prediction of acute kidney injury risk after percutaneous coronary intervention using machine learning techniques: A retrospective cohort study
Source: PLoS Med. 2018 Nov 27;15(11):e1002703. doi: 10.1371/journal.pmed.1002703 (PMC6258473; doi:10.1371/journal.pmed.1002703)
Supplement: S6 Table — (DOCX) [file pmed.1002703.s007.docx]

|  | **Variable selection method** | |
| --- | --- | --- |
| **Variable** | **Lasso regularization with logistic regression (No. times selected)** | **Permutation selection with XGBoost (No. times selected)** |
| Age | 100 | 100 |
| Sex | 0 | 0 |
| Admission source | 100 | 100 |
| Current/Recent smoker | 100 | 0 |
| Hypertension | 100 | 0 |
| Dyslipidemia | 100 | 0 |
| Family history of premature CAD | 100 | 0 |
| Prior MI | 0 | 0 |
| Prior heart failure | 100 | 100 |
| Prior valve surgery/procedure | 0 | 0 |
| Prior PCI composite | 100 | 0 |
| Prior CABG composite | 0 | 0 |
| Body mass index | 100 | 100 |
| Cerebrovascular disease | 100 | 0 |
| Peripheral arterial disease | 100 | 0 |
| Chronic lung disease | 100 | 0 |
| Diabetes mellitus composite | 100 | 100 |
| CAD presentation composite | 100 | 100 |
| Anginal classification w/in 2 weeks | 100 | 0 |
| Anti-anginal medication w/in 2 weeks composite | 100 | 0 |
| Beta blocker | 0 | 0 |
| Heart failure w/in 2 weeks composite | 100 | 100 |
| Cardiomyopathy or left ventricular systolic dysfunction | 0 | 0 |
| Cardiogenic shock w/in 24 hours | 100 | 100 |
| Cardiac arrest w/in 24 hours | 100 | 100 |
| Stress or imaging studies | 100 | 0 |
| IABP at the start of procedure | 100 | 0 |
| Other mechanical ventricular support at the start of procedure | 0 | 0 |
| PCI status | 100 | 100 |
| Pre-PCI ventricular ejection fraction | 100 | 100 |
| Pre-procedure GFR | 100 | 100 |
| Pre-procedure hemoglobin | 100 | 100 |

CAD indicates coronary artery disease; MI, myocardial infarction; PCI, percutaneous coronary intervention; CABG, coronary artery bypass grafting; NYHA, New York Heart Association; IABP, intra-aortic balloon pump; STEMI, ST elevation myocardial infarction.
