# Supplementary material for: Enhancing the prediction of acute kidney injury risk after percutaneous coronary intervention using machine learning techniques: A retrospective cohort study
Source: PLoS Med. 2018 Nov 27;15(11):e1002703. doi: 10.1371/journal.pmed.1002703 (PMC6258473; doi:10.1371/journal.pmed.1002703)
Supplement: S7 Table — XGBoost, extreme gradient boost. (DOCX) [file pmed.1002703.s008.docx]

|  | | **Model 8 predicted risk** | |
| --- | --- | --- | --- |
|  | | Lowest decile (<2.2%) | Highest decile (>15.3%) |
| N (%) | | 28,413 (10.0%) | 28,413 (10.0%) |
| Observed AKI rate | | 1.9% | 27.4% |
| **Mean predicted risk** | Model 1 (baseline) | 3.3% | 21.3% |
|  | Model 8 (XGBoost) | 1.9% | 27.1% |
| **Difference between mean predicted risk and observed rate** | Model 1 (baseline) | 1.4% | -6.2% |
|  | Model 8 (XGBoost) | 0.1% | -0.3% |

AKI indicates acute kidney injury.
