# Supplementary material for: Enhancing the prediction of acute kidney injury risk after percutaneous coronary intervention using machine learning techniques: A retrospective cohort study
Source: PLoS Med. 2018 Nov 27;15(11):e1002703. doi: 10.1371/journal.pmed.1002703 (PMC6258473; doi:10.1371/journal.pmed.1002703)
Supplement: S8 Table — (DOCX) [file pmed.1002703.s009.docx]

| **Characteristic** | **Development cohort (June 1, 2009-June 30, 2011)** | **Contemporary cohort (July 1, 2016-March 31, 2017)** | **P-value for difference** |
| --- | --- | --- | --- |
| N | 947,091 | 970,869 |  |
| No. AKI, % | 69,826 (7.37) | 72,954 (7.51) | <0.001 |
| Age, y | 64.8±12.2 | 65.7±12.1 | <0.001 |
| Female, n (%) | 311,013 (32.8) | 976,924 (31.9) | <0.001 |
| Race, n (%) |  |  |  |
| White | 838,467 (88.5) | 838,360 (86.4) | <0.001 |
| Black/African American | 74,820 (7.9) | 83,773 (8.6) | <0.001 |
| Admission source |  |  |  |
| Emergency department | 371,959 (39.3) | 457,819 (47.2) | <0.001 |
| Transfer from acute care facility | 174,032 (18.4) | 177,651 (18.3) | 0.167 |
| Body mass index, kg/m^2^ | 30.1±11.8 | 30.2±9.0 | <0.001 |
| Baseline GFR, ml/min/1.73m^2^ | 76.4±29.3 | 78.3±29.3 | <0.001 |
| GFR level, n (%) |  |  |  |
| Normal >=60 | 696,120 (73.5) | 726,322 (74.8) | <0.001 |
| Mild GFR 45 to 60 | 159,691 (16.9) | 152,858 (15.7) | <0.001 |
| Moderate GFR 30 to 45 | 71,091 (7.5) | 68,951 (7.1) | <0.001 |
| Severe GFR <30 | 20,189 (2.1) | 22,738 (2.3) | <0.001 |
| Anemia, n (%) | 33,988 (3.6) | 51,999 (5.4) | <0.001 |
| Hypertension, n (%) | 775,039 (81.8) | 803,612 (82.8) | <0.001 |
| Prior MI, n (%) | 282,352 (29.8) | 291,000 (30.0) | 0.015 |
| Prior heart failure, n (%) | 110,003 (11.6) | 147,249 (15.2) | <0.001 |
| Prior PCI, n (%) | 376,248 (39.7) | 375,220 (38.6) | <0.001 |
| Prior CABG, n (%) | 176,136 (18.6) | 163,113 (16.8) | <0.001 |
| Cerebrovascular disease, n (%) | 115,924 (12.2) | 129,517 (13.3) | <0.001 |
| Peripheral arterial disease, n (%) | 116,020 (12.3) | 114452 (11.8) | <0.001 |
| Chronic lung disease, n (%) | 144,177 (15.2) | 153,895 (15.9) | <0.001 |
| Diabetes mellitus, n (%) | 339,226 (35.8) | 380,097 (39.2) | <0.001 |
| CAD presentation, n (%) |  |  |  |
| No symptom, no angina | 83,317 (8.8) | 30,741 (3.2) | <0.001 |
| Symptom unlikely to be ischemic | 27,698 (2.9) | 17,050 (1.8) | <0.001 |
| Stable angina | 156,613 (16.5) | 100,121 (10.3) | <0.001 |
| Unstable angina | 342,774 (36.2) | 363,624 (37.5) | <0.001 |
| Non-STEMI | 181,890 (19.2) | 274,554 (28.3) | <0.001 |
| STEMI or equivalent | 154,525 (16.3) | 184,586 (19.0) | <0.001 |
| IABP before procedure, n (%) | 1,944 (0.2) | 2,426 (0.2) | <0.001 |
| Heart failure within 2 weeks, n (%) | 95,373 (10.1) | 138,302 (14.2) | <0.001 |
| Cardiogenic shock within 24 hours, n (%) | 17,219 (1.8) | 22,098 (2.3) | <0.001 |
| Cardiac arrest within 24 hours, n (%) | 17,250 (1.8) | 22,391 (2.3) | <0.001 |

Values are mean ± SD except as noted. AKI indicates acute kidney injury; GFR, glomerular filtration rate; CABG, coronary artery bypass grafting; MI, myocardial infarction; PCI, percutaneous coronary intervention; CAD, coronary artery disease; STEMI, ST-elevation myocardial infarction; GFR, glomerular filtration rate.
