# Supplementary material for: Enhancing the prediction of acute kidney injury risk after percutaneous coronary intervention using machine learning techniques: A retrospective cohort study
Source: PLoS Med. 2018 Nov 27;15(11):e1002703. doi: 10.1371/journal.pmed.1002703 (PMC6258473; doi:10.1371/journal.pmed.1002703)
Supplement: S9 Table — XGBoost, extreme gradient boost. (DOCX) [file pmed.1002703.s010.docx]

| **Model** | **Updating strategy** | **Data for building model** | **Data for re-calibration** | **Performance on evaluation set**  **Mean (95% confidence interval)** | | | | | |
| --- | --- | --- | --- | --- | --- | --- | --- | --- | --- |
|  |  |  |  | AUC | Brier score | Calibration intercept | Calibration slope | Reliability x10^-2^ | Resolution |
| Model 1 (Baseline model) | 1 | Development cohort | None | 0.753 (0.750-0.756) | 0.0627 (0.0625-0.0629) | -0.016 (  -0.012-  -0.015) | 1.062 (1.046-1.078) | 0.0151 (0.0138-0.0164) | 0.0057 (0.0055-0.0059) |
|  | 2 | Development cohort | Contemporary cohort updating set | 0.753 (0.750-0.756) | 0.0627 (0.0624-0.0629) | -0.002 (  -0.004-  -0.001) | 1.030 (1.009-1.051) | 0.0021 (0.0013-0.0029) | 0.0057 (0.0055-0.0059) |
|  | 3 | New cohort training set | None | 0.753 (0.750-0.756) | 0.0625 (0.0623-0.0628) | -0.003 (  -0.004-  -0.001) | 1.033 (1.013-1.054) | 0.0019 (0.0011-0.0028) | 0.0058 (0.0056-0.0060) |
|  | 4 | Development cohort and Contemporary cohort updating set | Contemporary cohort updating set | 0.753 (0.750-0.756) | 0.0626 (0.0623-0.0628) | -0.002 (  -0.004-  -0.001) | 1.031 (1.011-1.051) | 0.0021 (0.0014-0.0029) | 0.0058 (0.0056-0.0060) |
| Model 8 (XGBoost model) | 1 | Development cohort | None | 0.785 (0.783-0.788) | 0.0610 (0.0608-0.0612) | -0.013 (  -0.014-  -0.012) | 1.003 (0.994-1.011) | 0.0177 (0.0172-0.0182) | 0.0073 (0.0071-0.0074) |
|  | 2 | Development cohort | Contemporary cohort updating set | 0.785 (0.783-0.788) | 0.0609 (0.0607-0.0611) | -0.001 (  -0.002-0.000) | 1.013 (1.001-1.026) | 0.0004 (0.0000-0.0008) | 0.0073 (0.0071-0.0074) |
|  | 3 | New cohort training set | None | 0.789 (0.786-0.791) | 0.0605 (0.0603-0.0606) | -0.000 (  -0.001-0.000) | 1.008 (0.997-1.020) | 0.0004 (0.0000-0.0008) | 0.0075 (0.0074-0.0076) |
|  | 4 | Development cohort and Contemporary cohort updating set | Contemporary cohort updating set | 0.789 (0.786-0.791) | 0.0605 (0.0603-0.0607) | 0.003 (0.002-0.003) | 0.967 (0.955-0.979) | 0.0017 (0.0009-0.0025) | 0.0075 (0.0073-0.0076) |
