# Supplementary figures and images for: Enhancing the prediction of acute kidney injury risk after percutaneous coronary intervention using machine learning techniques: A retrospective cohort study
Source: PLoS Med. 2018 Nov 27;15(11):e1002703. doi: 10.1371/journal.pmed.1002703 (PMC6258473; doi:10.1371/journal.pmed.1002703)

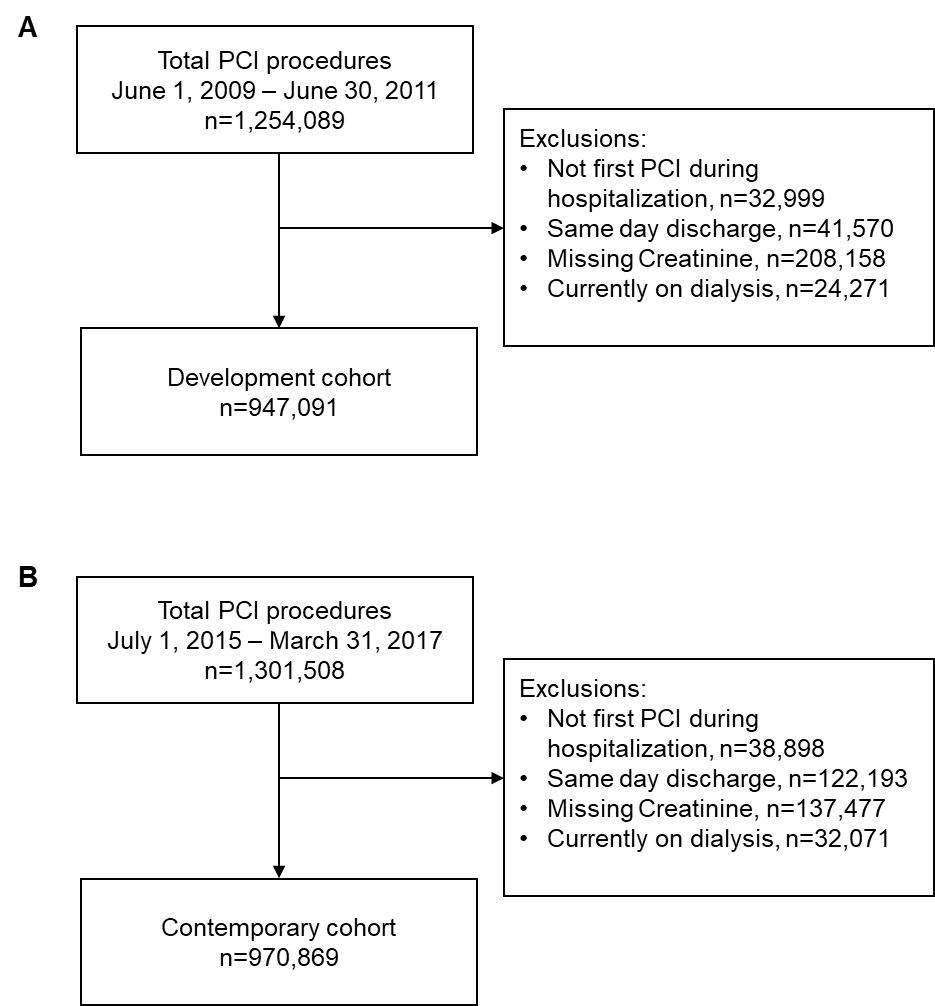

Supplement: S1 Fig — Flowchart of study participants for (A) the development cohort for the main analysis and (B) the contemporary cohort for temporal validation. PCI, percutaneous coronary intervention. (DOCX) [file pmed.1002703.s011.docx]

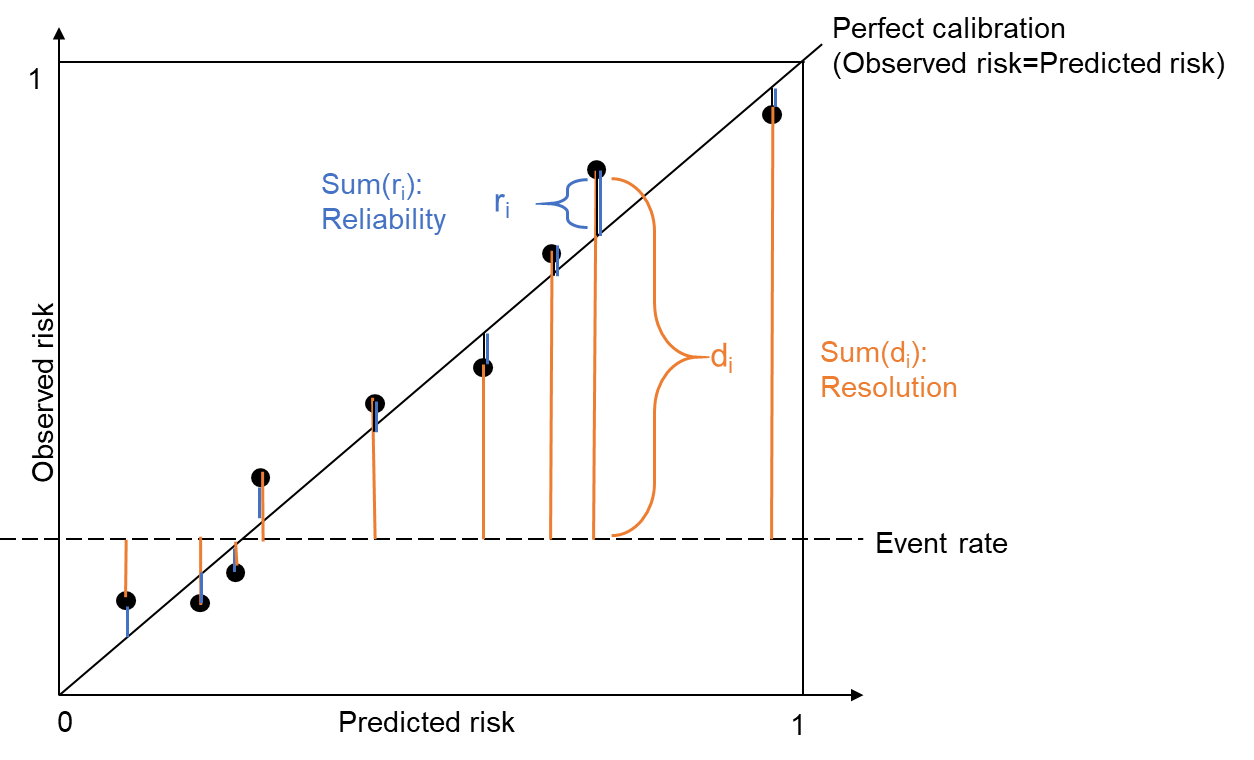

Supplement: S2 Fig — The x-axes of the points are the deciles of predicted risks, and the y-axes of the points are the observed event rate of the patients in each decile. (DOCX) [file pmed.1002703.s012.docx]

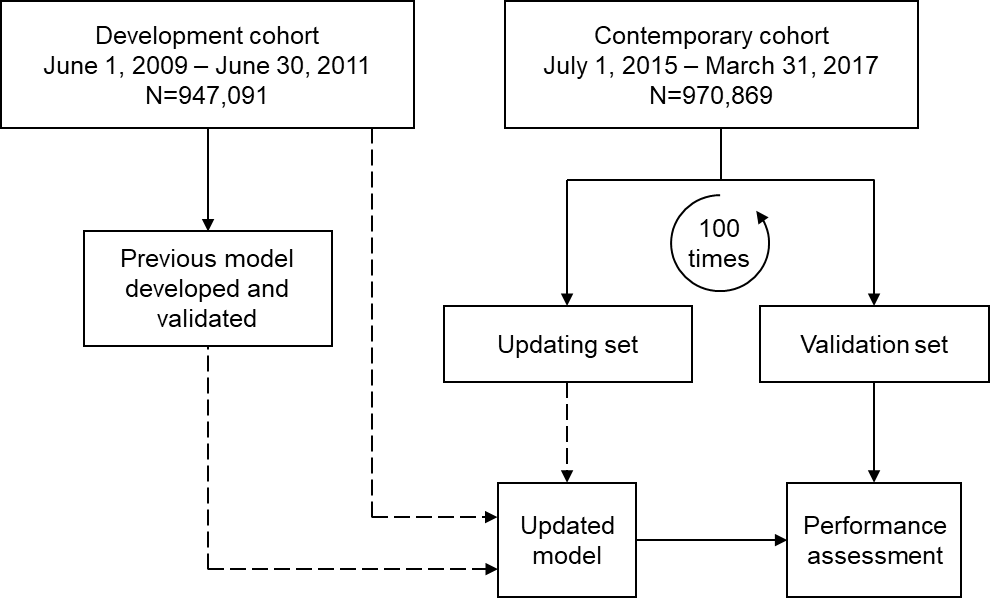

Supplement: S3 Fig — (DOCX) [file pmed.1002703.s013.docx]

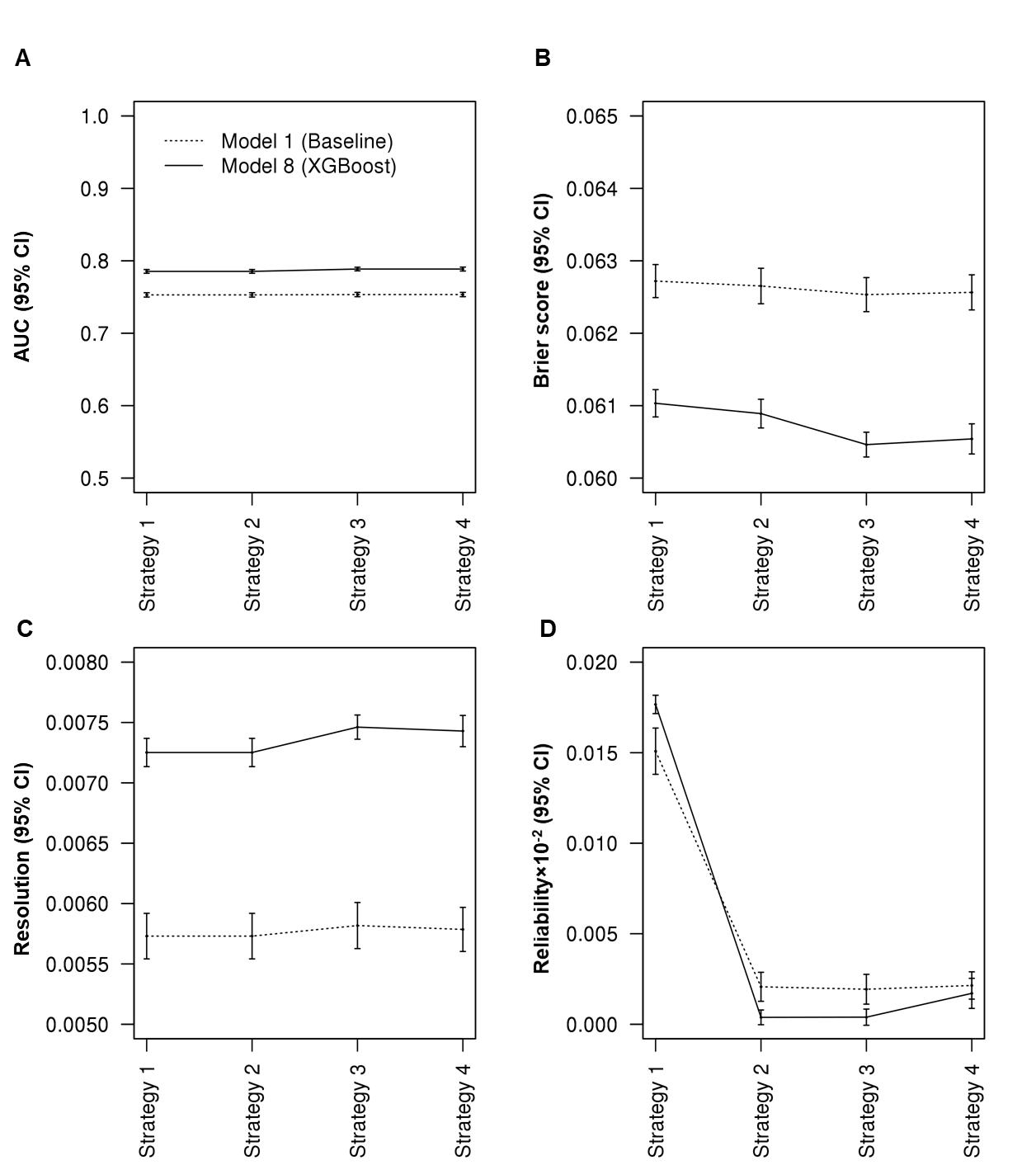

Supplement: S4 Fig — AUC, area under the receiver operating characteristics curve; CI, confidence interval; XGBoost, extreme gradient boost. (DOCX) [file pmed.1002703.s014.docx]
